# Phenotypic Variation in European Wild Pear (*Pyrus pyraster* (L.) Burgsd.) Populations in the North-Western Part of the Balkan Peninsula

**DOI:** 10.3390/plants11030335

**Published:** 2022-01-27

**Authors:** Antonio Vidaković, Zlatko Šatović, Katarina Tumpa, Marilena Idžojtić, Zlatko Liber, Valentino Pintar, Mira Radunić, Tonka Ninčević Runjić, Marko Runjić, Jakša Rošin, Daniel Gaunt, Igor Poljak

**Affiliations:** 1 Institute of Forest Genetics, Dendrology and Botany, Faculty of Forestry and Wood Technology, University of Zagreb, Svetošimunska cesta 23, HR-10000 Zagreb, Croatia; avidakovi@sumfak.hr (A.V.); ktumpa@sumfak.hr (K.T.); midzojtic@sumfak.hr (M.I.); danielgaunt12@gmail.com (D.G.); 2 Department for Seed Science and Technology, Faculty of Agriculture, University of Zagreb, Svetošimunska cesta 25, HR-10000 Zagreb, Croatia; zsatovic@agr.hr; 3 Centre of Excellence for Biodiversity and Molecular Plant Breeding, Svetošimunska cesta 25, HR-10000 Zagreb, Croatia; zlatko.liber@biol.pmf.hr (Z.L.); Mira.Radunic@krs.hr (M.R.); 4 Department of Biology, Faculty of Science, University of Zagreb, Marulićev trg 9a, HR-10000 Zagreb, Croatia; 5 Ministry of Economy and Sustainable Development, Institute for Environment and Nature, Nature Sector, Radnička cesta 80, HR-10000 Zagreb, Croatia; valentino.pintar@mingor.hr; 6 Institute for Adriatic Crops and Karst Reclamation, Put Duilova 11, HR-21000 Split, Croatia; Tonka.Nincevic@krs.hr (T.N.R.); Marko.Runjic@krs.hr (M.R.); Jaksa.Rosin@krs.hr (J.R.)

**Keywords:** European wild pear, morphometric analysis, leaf morphology, leaf variability, phenotypic plasticity, isolation by distance, isolation by environment, population diversity, population structure

## Abstract

Leaves play a central role in plant fitness, allowing efficient light capture, gas exchange and thermoregulation, ensuring optimal growing conditions for the plant. Phenotypic variability in leaf shape and size has been linked to environmental heterogeneity and habitat characteristics. Therefore, the study of foliar morphology in plant populations can help us to identify the environmental factors that may have influenced the process of species diversification. In this study, we used European wild pear (*Pyrus pyraster* (L.) Burgsd., Rosaceae) as a model species to investigate the phenotypic variability of leaves under different environmental conditions. Using leaf morphometric data from 19 natural populations from the north-western part of the Balkan Peninsula, a high level of variability among and within populations were found. Leaf traits related to leaf size were more variable compared to leaf shape traits, with both influenced by geographic and environmental factors. Consequently, patterns of isolation by environment (IBE) and distance (IBD) were identified, with IBE showing a stronger influence on leaf variability. Multivariate statistical analysis revealed that European wild pear populations from the north-western part of the Balkan Peninsula can be divided into two morphological clusters, consistent with their geographical distance and environmental conditions. Our results confirm a high level of phenotypic variability in European wild pear populations, providing additional data on this poorly studied species, emphasizing phenotypic plasticity as a major driver in the adaptation of this noble hardwood species to rapid climate change.

## 1. Introduction

Plants, as sessile organisms, express exceptional capacity to adjust their phenotypic and physiological characteristics to environmental heterogeneity they are exposed to [1]. This environmentally conditioned adaptation occurs through two possible evolutionary mechanisms: local adaptation and phenotypic plasticity [2]. The former refers to across-generations phenotypic and genetic differentiation under the environmental pressures, while latter concerns the within-generation phenotypic changes under the influence of different habitat and environmental conditions [3,4], with no genetic differentiation [5]. Phenotypic plasticity is therefore recognized as one of the key mechanisms in plants’ responses to various and fast-changing climatic conditions [1,6], as evolutionary mechanisms cannot always keep up with climate change [4]. These plastic responses are evident in a number of ecologically important traits, such as morphology, physiology, anatomy and phenology [7,8].

One of the most common outcomes of phenotypic plasticity is the great variation in leaf shape and size [9,10]. Accordingly, many studies have noted leaf phenotypic responses along gradients of various environmental factors, such as temperature [11,12,13,14], precipitation [15,16,17] and light [18,19,20]. The leaf size is reported to decline with decreasing annual temperature and precipitation, as well as under intensive light exposure. Decreasing leaf size in high light environments is necessary to avoid excessive radiation and thus prevent moisture loss and physical damage of the leaf structures [21]. On the other hand, leaf lamina tends to grow larger in lower irradiance, in order to maximize light capture [22]. Temperature affects both leaf size and shape, as lower temperatures were reported to positively correlate with leaf size and have caused more abundant leaf teeth and a greater degree of leaf dissection [12]. Hence, leaf shape, besides being genetically fixed, also shows susceptibility to environmental influences [9,23,24].

One of the genera that shows significant inter- and intra-species leaf phenotypic variability is *Pyrus* L. Since the European wild pear (*Pyrus pyraster* (L.) Burgsd.) is the most widespread of all pears in Europe, growing in a wide range of suitable habitats, it was selected as a model-species to examine the phenotypic variability conditioned by environmental and habitat heterogeneity. Its natural distribution covers the area of southern, central, and western Europe [25,26], with the species occurring in a scattered distribution pattern, as solitary trees or in small groups on the forest edges and on abandoned agricultural or grazing lands [27]. It grows as a small to medium-sized deciduous tree, characterized by grey, scaly bark and thin, angular and often thorny branches. Leaves of this species are phenotypically diverse, round to ovoid, finely serrated, less often with complete leaf edges, varying greatly even on the individual level. White colored, insect-pollinated flowers, with an unpleasant smell, appear from April through May. The fruit is up to 3 cm, a big pome, subglobose, turbinate or pyriform, with the peduncle of the approximately same length [25,28]. Pomes serve as an important food source for various mammals, including badgers, foxes and wild boars [29].

European wild pear is a light-demanding species that can grow on almost all soil types and textures. It can be found on warm and well saturated but drained soils, both nutrient and base-rich, mostly carbonate but occasionally non-carbonate [25]. The species usually occupies extreme or marginal sites [30], growing in thickets and open temperate forests. Its broad natural range stretches from lowlands to hills and sometimes into the mountains, appearing within the altitude range of 0–1520 m above sea level, but avoiding frost susceptible areas. European wild pear is primarily a floristic element of mixed oak and hornbeam forests, open, thermophilic mixed-oak forests and riparian forests [25]. This usually includes extremely dry locations or, on the other hand, repeatedly flooded lowland areas [31]. Considering the wide ecological amplitude of the species observed through the lens of the principles of local adaptation and phenotypic plasticity, great phenotypic variability of the European wild pear comes as no surprise [32,33]. As a result, many lower European wild pear taxa were described in the past [27,34,35,36]. Nowadays, however, high variability is attributed to local adaptation and/or phenotypic plasticity, indicating that those lower taxa are simply a part of intraspecific variability conditioned by local habitat and climatic conditions [37].

In this study, we aimed to examine the phenotypic variability of European wild pear based on leaf material from 19 natural populations from the north-western part of the Balkan Peninsula. The aim was to study how leaf phenotypic traits vary among and within European wild pear populations in response to different in situ environmental conditions and to test the influence of isolation by distance (IBD) and isolation by environment (IBE) on the phenotypic variability of the species. It is expected that the ability of the species to adapt to local conditions will cause pronounced leaf phenotypic variations among and within European wild pear populations under various environmental conditions.

## 2. Materials and Methods

### 2.1. Plant Material and Study Area

**Figure 1:**
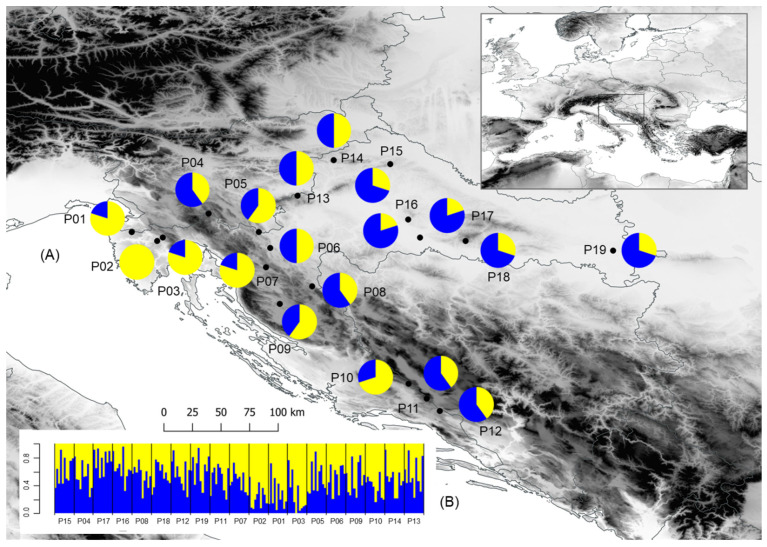
Results of the multivariate statistical methods and locations of the 19 sampled *Pyrus pyraster* populations, based on 10 morphological leaf traits. (**A**) Geographical distribution of two groups of populations detected from K-means clustering method (the proportions of the membership of each population in each of the defined clusters are color-coded: cluster A—yellow, cluster B—blue); and (**B**) barplot with posterior probabilities of classification of each individual into each group from the results of the classification discriminant analysis. Populations: P01—Kuberton; P02—Hum; P03—Lupoglav; P04—Kozji vrh; P05—Lukovdol; P06—Ogulin; P07—Brinje; P08—Plitvička jezera; P09—Perušić; P10—Rumin; P11—Voštane; P12—Studenci; P13—Žumberak; P14—Strahinščica; P15—Kalnik; P16—Moslavačka gora; P17—Lipovljani; P18—Psunj; P19—Vinkovci.

A total of 190 European wild pear trees from 19 natural populations were sampled across the north-western part of the Balkan Peninsula, including various environmental conditions (Table S1, Figure 1). The study area encompasses the conditions of the continental climate in the east to the sub-Mediterranean climate in the west, which is also a marginal area of natural distribution and ecological niche of the European wild pear. Several populations in the southern part of the study area covered extreme habitats characterized by very high insolation, well-drained, dry soils, and summer droughts.

### 2.2. Sampling Method

Samples for morphometric analyses were collected during the vegetation season of 2021, from 10 adult, sexually matured trees in each population. Special attention was paid to avoid trees that we suspected of being hybrids with cultivated pears. Descriptions from the literature were used for accurate identification. European wild pears were identified by the presence of thorns, round to ovoid leaves that were never thickly felted, and round fruits [38,39,40]. As previously recorded by Paganová [36], leaves on short shoots are the most representative ones in terms of size and shape. Accordingly, short, sunlit shoots were collected from each tree, stored in plastic bags and transferred to the laboratory, where 30 fully developed and undamaged leaves were picked from each tree. Leaves were then pressed, measured, stored in paper envelopes and deposited in the herbarium at the Faculty of Forestry and Wood Technology of the University of Zagreb (DEND).

### 2.3. Studied Phenotypic Traits

Leaf phenotypic traits were measured using the Winfolia program [41] with the accuracy of 0.1 mm. A total of 10 phenotypic traits were measured, of which seven refer to the leaf size: leaf area (LA); perimeter (PE); leaf length (LL); maximum leaf width (MLW); leaf length, measured from the leaf base to the point of maximum leaf width (PMLW); leaf blade width at 90% of leaf blade length (LWT); and petiole length (PL); and three concerning leaf shape: form coefficient (FC) and leaf angles LA10 and LA25. LA10 and LA25 are traits describing the base of the leaf blade by expressing the angles closed by the main leaf vein (the center of leaf blade) and the line connecting the leaf blade base to a set point on the leaf margin, at 10% (LA10) and 25% (LA25) of the total leaf blade length.

### 2.4. Environmental Data

Data of the average climatic conditions for the period from 1970 to 2000, in the area of the studied populations, was obtained from the WorldClim 2 database with a spatial resolution close to a square kilometer [42]. The bioclimatic variables represent annual trends, seasonality and extreme or limiting environmental factors, useful when quantifying the effects of environmental conditions and climate changes on species distributions and phenotypic variability [43]. All 19 bioclimatic variables were included in the analysis (Table S1): BIO1 (Annual Mean Temperature); BIO2 (Mean Diurnal Range (Mean of monthly (max temp–min temp)); BIO3 (Isothermality (BIO2/BIO7) (×100)); BIO4 (Temperature Seasonality (standard deviation ×100)); BIO5 (Max Temperature of Warmest Month); BIO6 (Min Temperature of Coldest Month); BIO7 (Temperature Annual Range (BIO5-BIO6)); BIO8 (Mean Temperature of Wettest Quarter); BIO9 (Mean Temperature of Driest Quarter); BIO10 (Mean Temperature of Warmest Quarter); BIO11 (Mean Temperature of Coldest Quarter); BIO12 (Annual Precipitation); BIO13 (Precipitation of Wettest Month); BIO14 (Precipitation of Driest Month); BIO15 (Precipitation Seasonality (Coefficient of Variation)); BIO16 (Precipitation of Wettest Quarter); BIO17 (Precipitation of Driest Quarter); BIO18 (Precipitation of Warmest Quarter); BIO19 (Precipitation of Coldest Quarter). The variables were selected to describe the environmental characteristics of the studied populations and for the calculation of the environmental distance matrix.

### 2.5. Population Phenotypic Diversity

Phenotypic diversity of measured leaf size and shape traits in the studied populations was examined using descriptive statistics (arithmetic mean, standard deviation, minimum and maximum value, and coefficient of variation) and hierarchical analysis of variance. Using the mentioned statistical methods, we quantified leaf traits values, their range of variation as well as levels of variability on both among- and within-population levels. The analysis factors were populations and trees within populations.

To assess the possibility of conducting multivariate statistical analyses and parametric tests, the symmetry, unimodality and homoscedasticity of data were verified [44]. Assumptions of normality were checked using the Shapiro–Wilk test, and the assumption of homogeneity of variance using Levene’s test.

### 2.6. Population Structure

To identify the divergence and structure of the studied populations, multivariate statistical methods were used. The correlations between the studied leaf traits were verified using Pearson’s correlation coefficient to avoid the most redundant ones [44].

Using the K-means clustering method, based on the data for all 10 phenotypic leaf traits, we determined the number of clusters, which optimally illustrated the differentiation between the studied populations. Populations were considered to belong to one cluster or to be of a mixed origin based on whether a specific population proportion was greater than or equal to 0.7 (one cluster) or less than 0.7 (mixed origin) [45,46]. To further examine the structure between studied populations, we used the unweighted pair group method with arithmetic mean (UPGMA) clustering method to generate a dendrogram from a Euclidean distance matrix based on 10 phenotypic traits. Standardization of traits to zero mean and unit standard deviation was performed prior to K-means and cluster analysis. Principal component (PC) analysis was used to calculate the principal components across all individuals, and all studied morphometric traits. A biplot was constructed using two principal components showing the analyzed individuals and traits. In addition, analysis of discrimination was performed in order to evaluate the utility and importance of measured leaf traits by determining which were most useful in discriminating the populations and to eliminate possible redundant variables. Using the classification discriminant analysis, we determined the proportion of individuals accurately classified into the clusters detected by the K-means clustering method. Posterior probabilities of classification of each individual into studied groups from the results of the classification discriminant analysis were presented with a barplot. In performing all statistical analyses, we used software packages STATISTICA version 13 [47] and R v.3.4.3 [48].

### 2.7. Correlation between Environmental, Geographic and Phenotypic Data

Correlations between multitrait differences among populations were evaluated by performing the simple Mantel test, first used for such purposes by Sokal [49]. It is considered to be the universal method for testing the relationship between multivariate data sets expressed as dissimilarity matrices in biological problems, commonly used to quantify the degree of difference between individuals, populations or species [50]. In our study, three dissimilarity matrices were calculated to describe differences between the studied European wild pear populations: (1) phenotypic differences as squared Mahalanobis distances between the pairs of populations; (2) environmental distances as the Euclidian distances between the population means for the first three factors of the principal component analysis; and (3) geographic distance from the latitude and longitude of the sampling site. In addition, a three-way Mantel test was applied between the matrix of environmental differences and the matrix of pairwise phenotypic differences, while accounting for geographical distances among studied populations. The significance level was assessed after 10,000 permutations as implemented in NTSYS-pc Ver. 2.21L [51]. In addition, the relationships between individual leaf phenotypic traits and longitude, latitude and bioclimatic variables were tested using Pearson’s correlation coefficient.

## 3. Results

### 3.1. Climate Differences among Sampling Sites

We found pronounced differences in climate data among sampling sites, which is clearly visible from the results of the principal component analysis (Table S2, Figure S1). The first (PC1) and the second principal component (PC2) explained 42.36% and 26.58% of total variance in the studied data set, respectively. The first principal component was highly negatively correlated with BIO12 (Annual Precipitation), BIO13 (Precipitation of Wettest Month), BIO16 (Precipitation of Wettest Quarter) and BIO19 (Precipitation of Coldest Quarter). This principal component clearly distinguished the four eastern populations that shared low precipitation values from the other studied populations. The second principal component was highly positively correlated with three bioclimatic variables: BIO2 (Mean Diurnal Range), BIO9 (Mean Temperature of Driest Quarter) and BIO15 (Precipitation Seasonality); and negatively with BIO18 (Precipitation of Warmest Quarter). Of those, the former three variables best explained variation in populations Rumin (P10), Voštane (P11) and Studenci (P12), while BIO18 influenced the populations Kuberton (P01), Lupoglav (P03) and Ogulin (P06).

### 3.2. Phenotypic Traits and Populations’ Diversity

The distribution frequency of the examined traits was normal or only slightly left- or right-biased (data not shown), which enabled further statistical analysis.

Almost all traits proved to be in a statistically significant positive or negative correlation with each other, with few exceptions (data not shown). Namely, no significant correlation was recorded between the following pairs: LA with FC and LA25, PE with LA10 and LA25, LL with LWT and LA10 and LA25 with PL. However, all of the r values were lower than 0.95, enabling their utilization in further multivariate statistical methods.

The results of descriptive statistics for all studied populations together are shown in Table 1, and individually for each population in Table S3. From the obtained results it is noticeable that the most variable leaf phenotypic traits with the coefficient of variation (CV) above 30% were leaf area (LA), leaf width top (LWT) and petiole length (PL). On the other hand, the least variable leaf traits were those related to angles closed by the main leaf vein and the line defined by the leaf blade base and a point on the leaf margin (LA10 and LA25), with a CV lower than 10%. All other traits showed intermediate values of CV (13.70–23.84%). Accordingly, it was evident that leaf traits related to its shape were significantly less variable compared to the ones regarding its size. Observing the results for each of the studied populations separately (Table S3), it was clear that all phenotypic features have the lowest value in one of the three sub-Mediterranean populations (Kuberton (P01), Hum (P02) and Lupoglav (P03)). Among them, population Lupoglav (P03) had most of the minimum values (LA, MLW, LWT, LA10 and LA25), and was, at the same time, the most variable one. The second most variable was the population Ogulin (P06). The largest leaf area and maximum leaf width were specific to the population Kalnik (P15), while the highest values for leaf angles LA10 and LA25 were noted for the population Ogulin (P06). Among all of the studied populations, Studenci (P12) was the least variable one, accompanied by the other two southern populations, Voštane (P11) and Rumin (P10), with the lowest coefficient of variation for five leaf traits (LA, PE, LL, PMLW and LWT). Overall, a clear differentiation in leaf size was observed between western and eastern populations, displaying the smallest and the largest leaves, respectively, while southernmost and Dinaric populations showed intermediate values. Such geographical differentiation co-occurs with differences in habitat, population size and climatic conditions.

According to results of the hierarchical analysis of variance (ANOVA) (Table 2), statistically significant differences were present both within and among studied populations for almost all measured leaf traits (*p* < 0.01). The only exceptions in such distribution of data were present on the inter-population level for form coefficient (FC; *p* = 0.15) and leaf length, measured from the leaf base to the point of maximum leaf width (PMLW; *p* = 0.09). Among-population variability was accountable for the smallest percent of the total variability in all phenotypic traits, followed by within-population variability. Accordingly, the component of error was responsible for the largest share of the total variability for almost all leaf traits, except LWT, LA10 and LA25, which show the largest within-population variability.

### 3.3. Population Structure

**Figure 2:**
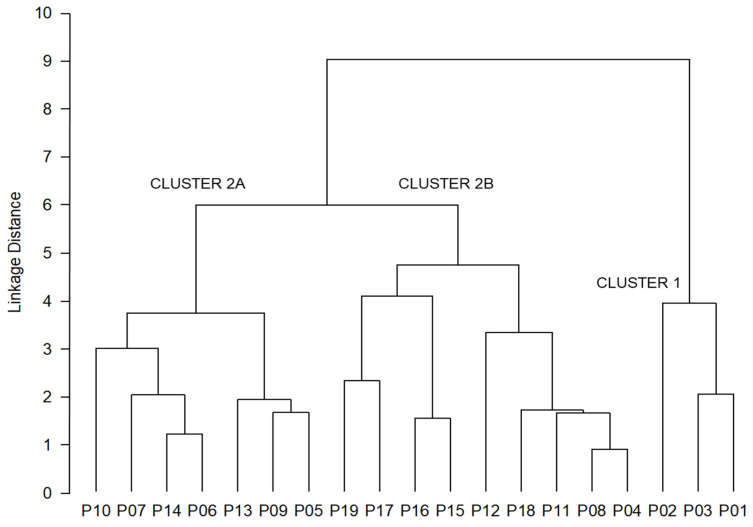
Tree diagram of the 19 *Pyrus pyraster* populations studied. The unweighted pair-group method with arithmetic mean (UPGMA) was used to join the clusters, and the Euclidean distance to define the distance between the studied populations based on 10 phenotypic traits. Populations: P01—Kuberton; P02—Hum; P03—Lupoglav; P04—Kozji vrh; P05—Lukovdol; P06—Ogulin; P07—Brinje; P08—Plitvička jezera; P09—Perušić; P10—Rumin; P11—Voštane; P12—Studenci; P13—Žumberak; P14—Strahinščica; P15—Kalnik; P16—Moslavačka gora; P17—Lipovljani; P18—Psunj; P19—Vinkovci.

The results of the conducted K-means clustering method revealed optimal division of the studied populations into two groups (Figure 1). The first cluster (A) consisted of the three sub-Mediterranean populations of Kuberton (P01), Hum (P02) and Lupoglav (P03), in addition to populations Brinje (P07) and Rumin (P10). On the other hand, eastern lowland floodplain populations Klanik (P15), Moslavačka gora (P16), Lipovljani (P17), Psunj (P18) and Vinkovci (P19) formed the second cluster (B). The remaining populations were a mixture of these two clusters, with some being closer to the first cluster (Lukovdol (P05) and Perušić (P09)) and four populations closer to the second one (Kozji vrh (P04), Plitvička jezera (P08), Voštane (P11) and Studenci (P12)). Three populations, however, were evenly classified into both clusters: Ogulin (P06), Žumberak (P13) and Strahinščica (P14). A similar distribution of populations was also revealed by the UPGMA clustering method (Figure 2). Namely, populations also diverged into two clusters, one of which further divided into two subclusters. The first, smaller cluster (1) consisted of all three western sub-Mediterranean populations (Kuberton (P01), Hum (P02) and Lupoglav (P03)), whereas the second, bigger cluster (2) further diverged into two distinct subclusters. The first subcluster (2A) contained the Dinaric and northern populations of Brinje (P07), Ogulin (P06), Perušić (P09), Lukovdol (P05), Žumberak (P13), Strahinščica (P14) and Rumin (P10). The second subcluster (2B) included eastern populations of Vinkovci (P19), Lipovljani (P17), Moslavačka gora (P16), Kalnik (P15) and Psunj (18), together with the southern populations of Studenci (P12) and Voštane (P11), as well as Dinaric populations of Plitvička jezera (P08) and Kozji vrh (P04). The grouping pattern of the southern and Dinaric population with the eastern populations, revealed by UPGMA clustering, was observed in clustering by K-means method as well.

**Figure 3:**
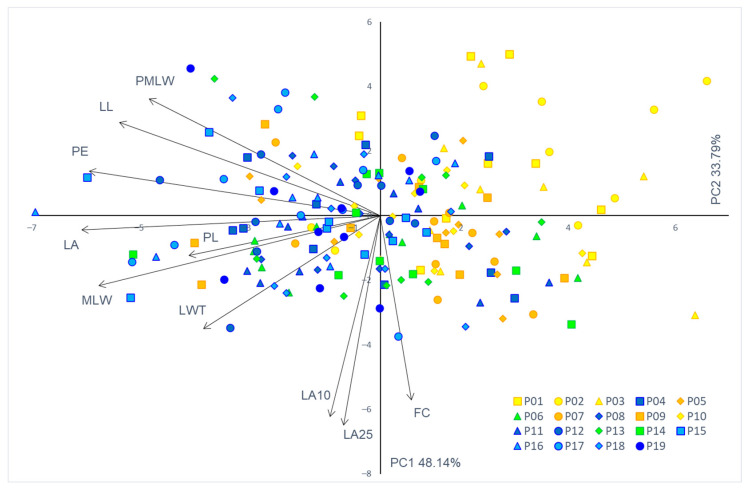
Biplot of the principal component (PC) analysis based on 10 leaf phenotypic traits in the studied *Pyrus pyraster* populations. Leaf phenotypic traits: LA—leaf area; PE—perimeter; LL—leaf length; MLW—maximum leaf width; PMLW—leaf length, measured from the leaf base to the point of maximum leaf width; LWT—leaf width top; PL—petiole length; FC—form coefficient; LA10—angle closed by the main leaf vein (the center of the leaf blade) and the line connecting the leaf blade base to a set point on the leaf margin at 10% of total leaf blade length; and LA25—angle closed by the main leaf vein (the center of the leaf blade) and the line connecting the leaf blade base to a set point on the leaf margin at 25% of total leaf blade length. Populations: P01—Kuberton; P02—Hum; P03—Lupoglav; P04—Kozji vrh; P05—Lukovdol; P06—Ogulin; P07—Brinje; P08—Plitvička jezera; P09—Perušić; P10—Rumin; P11—Voštane; P12—Studenci; P13—Žumberak; P14—Strahinščica; P15—Kalnik; P16—Moslavačka gora; P17—Lipovljani; P18—Psunj; P19—Vinkovci.

The first two components from the PC analysis of the phenotypic traits explained 48.14% and 33.79% of the total variation, respectively (Table 3). PC1 was highly negatively correlated with leaf area (LA), perimeter (PE), maximal leaf width (MLW), and leaf length (LL), i.e., variables related to the leaf dimensions. On the other hand, PC2 was highly negatively correlated with leaf angles (LA10 and LA25) and form coefficient (FC), i.e., variables related to the leaf shape. An overlap in the PC diagram was observed between the studied populations (Figure 3).

In order to determine morphological traits that had the highest discrimination power between the 19 studied populations, discrimination analyses were performed. The PE and FC were highly redundant. Therefore, PE was omitted from subsequent analyses. Overall results of the discrimination analyses based on nine morphological leaf traits showed statistical significance in discriminating between studied populations (Wilks’ λ = 0.092; F (162.1) = 2.831; *p* < 0.00001).

According to the partial Wilks’ λ values, the best discriminating factors between the studied populations were LA (partial Wilks’ λ = 0.744; *p* = 0.0001) and PL (partial Wilks’ λ = 0.746; *p* = 0.0001). The remaining factors all demonstrated similar partial lambda values, within the range of 0.835–0.921, with only two traits not demonstrating statistically significant values: FC (0.886) and PMLW (0.921) (Table S4). Traits both related to leaf size and shape proved to be statistically significant in discriminating between the populations.

**Figure 4:**
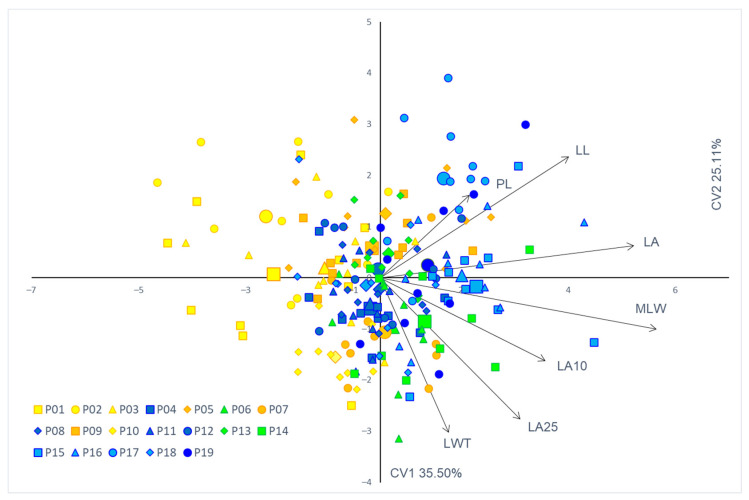
Discriminant analysis of 19 European wild pear populations based on seven leaf phenotypic traits that were the most useful for maximum discrimination between studied populations. Each individual tree is indicated by a small sign, while the population barycenters are represented by larger ones. Leaf phenotypic traits: LA—leaf area; LL—leaf length; MLW—maximum leaf width; LWT—leaf width top; PL—petiole length; LA10—angle closed by the main leaf vein (the center of the leaf blade) and the line connecting the leaf blade base to a set point on the leaf margin at 10% of total leaf blade length; and LA25—angle closed by the main leaf vein (the center of the leaf blade) and the line connecting the leaf blade base to a set point on the leaf margin at 25% of total leaf blade length. Canonical discriminant variate (CV).

For seven variables and 19 groups defined in the canonical analysis, seven canonical discriminant variates were defined. Figure 4 presents projections of canonical variables for canonical discriminant variates 1 (CV1) and 2 (CV2). CV1 accounted for 35.50% of the variation between the examined populations, whereas CV2 explained 25.11% of the total variation. Despite significant overlap between studied populations in morphospace, CV1 showed great influence in discrimination between the western, sub-Mediterranean populations (P01—Kuberton; P02—Hum; P03—Lupoglav) characterized by smaller leaves, and eastern populations (P15—Kalnik; P16—Moslavačka gora; P17—Lipovljani; P19—Vinkovci), characterized by the largest leaves measured in the research.

The proportion of individuals correctly classified into the groups detected by K-means clustering method was determined using classification discriminant analysis. The discriminant function based on seven morphometric traits showed classification success of 70.5%. European wild pear individuals were accurately classified into the first cluster (A) or the second cluster (B) in 72.0% and 68.9% of cases, respectively. The barplot with posterior probabilities of classification of each individual into each group from the results of the classification analysis of discrimination is shown in Figure 1B. Populations most accurately classified into the first cluster (A) were Kuberton (P01), Hum (P02), Lupoglav (P03) and Rumin (P10), with the classification accuracy of 80—90%. On the other hand, populations Plitvička jezera (P08), Studenci (P12), Kalnik (P15), Moslavačka gora (P16), Lipovljani (P17) and Vinkovci (P19) had the largest number of accurately classified trees into the second cluster (B), with the accuracy of their classification ranging between 70 and 100%.

### 3.4. Isolation by Distance (IBD) and Isolation by Environment (IBE)

**Figure 5:**
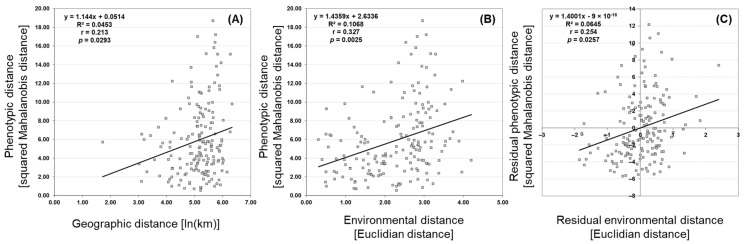
Isolation by distance (IBD) and isolation by environmental distance (IBE) in European wild pear populations. Scatter plots of simple and partial Mantel’s tests showing the relationships between (**A**) geographic and phenotypic distances (r = 0.213, *p* = 0.0293, R^2^ = 0.0453); (**B**) environmental and phenotypic distances (r = 0.327, *p* = 0.0025, R^2^ = 0.1068); and (**C**) residual environmental and phenotypic distances (r = 0.254, *p* = 0.0257, R^2^ = 0.0645) by taking into account the geographic distances among 19 European wild pear populations.

The analyzed populations showed significant correlations both between phenotypic and geographic distances (isolation by distance (IBD), (r = 0.213, *p* = 0.0293, R^2^ = 0.0453) (Figure 5A) and even higher correlations between phenotypic and environmental distances (isolation by environment (IBE), (r = 0.327, *p* = 0.0025, R^2^ = 0.1068) (Figure 5B). The correlation between phenotypic and environmental distances remained significant (r = 0.254, *p* = 0.0257, R^2^ = 0.0645) even after accounting for the effect of geographical distance in a three-way Mantel test (Figure 5C), confirming that the differences in environment influenced the structuring of the phenotypic diversity of European wild pear populations. When observing the residual environmental distance (Figure 5C), R^2^ indicated 6.45% of total variability to be attributed to the environmental factors alone.

In order to determine which specific variables correlated with each of the bioclimatic variables and geographic coordinates, Pearson’s correlation coefficient was calculated (Table S5). Statistically significant positive correlations were determined between temperature-related bioclimatic variables BIO4 (Temperature Seasonality), BIO7 (Temperature Annual Range) and BIO8 (Mean Temperature of Wettest Quarter) and the following leaf traits: LA, PE, LL and MLW. On the other hand, significantly high negative correlations were confirmed between variables BIO6 (Min Temperature of Coldest Month), BIO12 (Annual Precipitation), BIO13 (Precipitation of Wettest Month), BIO14 (Precipitation of Driest Month), BIO16 (Precipitation of Wettest Quarter), BIO17 (Precipitation of Driest Quarter) and BIO19 (Precipitation of Coldest Quarter) and LA, PE, LL, MLW and PMLW. Leaf traits related to its shape (LA10 and LA25) were positively correlated with latitude, BIO4 and BIO7 and negatively with BIO6 and BIO11 (Mean Temperature of Coldest Quarter). Leaf traits FC and PL, and bioclimatic variables BIO2 (Mean Diurnal Range), BIO3 (Isothermality), BIO5 (Max Temperature of Warmest Month), BIO9 (Mean Temperature of Driest Quarter), BIO15 (Precipitation Seasonality) and BIO18 (Precipitation of Warmest Quarter) were not correlated with any of the other variables.

## 4. Discussion

European wild pear is a phenotypically variable species for which, in the past, two subspecies and many varieties and forms have been described [35]. Currently, many of those taxa are considered to be synonyms, such as *P*. *communis* var. *pyraster* L., *P*. *communis* var. *achras* (Gaertn.) Wallr., *P*. *communis* subsp. *achras* (Gaertn.) Asch. et Graebn., *P*. *communis* var. *sylvestris* Lam. et DC, *P*. *pyraster* subsp. *achras* (Gaertn.) Terpø and *P*. *pyraster* f. *rotundifolia* (Gillot) Terpø. Such a large number of previously described forms and varieties certainly indicates great phenotypic variability of the species. Reported values for leaf length of European wild pear differ between various authors, with a summarized range of 2–8 cm [25,27,31,52,53]. According to the same authors, leaf width is within the range of 1.5–5 cm, while the petiole is described to be 1.5–7 cm long. Our results fall within previously mentioned data and are in the range of 1.6–6.9, 1.1–5.7 and 0.6–8 cm, for leaf length, leaf width and petiole length, respectively.

It is reported that leaf shape is extremely variable among pear species, and that three distinct leaf shapes can be identified within this genus [54]: broad, cordate leaves (referred to as “broad” leaves); narrow, lanceolate leaves (“narrow”); and leaves intermediate in shape between the other two forms (“intermediate”). Broad leaves are generally indicative of plants inhabiting mesophytic habitats and narrow of xerophytic habitats [54,55]. Leaf shape of European wild pear is usually described as simple, round to ovoid, with a shortly pointed apex and a rounded or shallow heart-shaped base [39,53,56], which classifies this pear species as a “broad-leaf” type. Mean form coefficient in our research was 0.9, confirming round to ovoid leaf shape. However, coefficient of variation in this study indicates significant and moderate leaf shape variation. In all studied populations, “intermediate” leaf form individuals with form coefficient 0.5–0.6, were recorded. We assume that this within- and among-population variation in leaf shape is a consequence of European wild pear genetic diversity and plastic response, within individual plants, suggesting that leaves play a significant role in adaptation to local environmental conditions [54].

When observing the descriptive data, leaf traits related to the size proved to be highly variable, with the coefficient of variation ranging from 19.18 to 36.06%. However, this is not surprising since leaves are known to play a crucial role in plant–environment interactions, i.e., light capture, gas exchange and thermoregulation [9,23,57]. Consequently, adjustment of leaf morphology is a common mechanism employed by the plants in conditions of varying sunlight, water availability or mechanical stressors [58]. Leaf size and petiole length are in close relation to phyllotaxy, determining the success of light capture [59], causing their high variability [10]. This was also confirmed by other authors who studied leaf trait diversity for other tree species, such as *Alnus incana* (L.) Moench [46], *Castanea sativa* Mill. [60], *Fagus sylvatica* L. [61,62], *Prunus avium* (L.) L. [63], *Pyrus mamorensis* Trab. [64], *P*. *spinosa* Forssk. [65], *Sorbus torminalis* (L.) Crantz [66] and *Ulmus minor* Mill. [67]. However, the high variability in leaf size and shape in other pear species [64,65,68] did not result in a large number of the described lower taxa, which casts doubt on the justification of so many described varieties and forms of European wild pear.

High diversity of European wild pear populations was revealed in this study, both at within- and among-population levels. The most variable populations were Lupoglav (P03) and Ogulin (P06), where the largest number of individuals was observed. Generally, larger populations are predicted to have lower inbreeding and genetic drift rates, resulting in higher genetic variation [69], which can only be maintained in genetically heterogeneous, cross-fertilizing populations [70]. Consequently, those populations are usually characterized with higher phenotypic diversity. On the other hand, the lowest variability was recorded in three southernmost, hinterland populations located on forest glades at high altitudes. Because growing conditions are suboptimal for European wild pear in this area, the species is quite rare, forming very small populations of 10–15 individuals, mainly on the edges of meadows where more water is retained than in the surrounding karst area. In contrast, crab apple (*Malus sylvestris* (L.) Mill., Rosaceae) populations, found in the same area, have demonstrated the greatest genetic variability in Europe and Caucasus [71]. These results were attributed to the fact that this part of the Balkan Peninsula served as glacial refugia for a number of temperate species which, faced with the accumulation and growth of ice cover over the northern part of the continent, retreated and remained in southern Europe. As it is reasonable to assume that European wild pear survived glaciation at least partially in the same areas as the wild apple, great diversity of its populations in this area is to be expected. This research, however, shows the lowest variability in the area close to the assumed refugial region. This indicates that phenotypic variability is potentially affected by phenotypic plasticity and adaptation of European wild pear populations, as well as by the loss of genotypes under various environmental and anthropogenic pressures. On the other hand, we cannot exclude the possibility that those southernmost populations are characterized with unique alleles and specific genetic diversity. Furthermore, although they are geographically quite close, they were highly divergent when leaf shape and dimensions were taken into consideration.

Overall, our results suggest that European wild pear populations from the north-western part of the Balkan Peninsula can be divided into two morphological clusters, consistent with their geographical distance and environmental conditions to a certain extent. However, the influence of genetics, microclimate and pedology cannot be excluded when observing the phenotypic diversity of the populations. Despite being exposed to high temperatures and precipitation levels, the westernmost populations surprisingly had the smallest leaf dimensions. Such unexpected results were also confirmed in other woody species: *Fagus sylvatica* [17], *Castanea sativa* [60] and *Ternstroemia lineata* DC. [72]. Meier and Leuschner [17] noted that beech leaf expansion and stand leaf area along precipitation gradient are, in addition to water availability, controlled by spring temperature and possibly nitrogen supply. Furthermore, these two factors increased in value towards drier sites, thus overlaying any negative effect of water shortage on leaf development. Although characterized with the highest precipitation values, the small-leaf populations of European wild pear from the western part of the study area are probably the result of phenotypic plasticity and adaptation to karstic, well drained soils, flysch and high thermophilicity of the area. On the other hand, the eastern populations, characterized by high temperatures but low precipitation levels had the largest leaves. Here, populations Kalnik (P15) and Moslavačka gora (P16) stand out the most with their dimensions. These two populations are located at slightly higher altitudes and are part of the very rich and highly productive mesophilic forests that dominate this area. In these populations, deep and well saturated soils, covered with thick leaf litter layer, provide needed humidity which, in addition to warm temperature, aids leaf growth. The rest of the eastern populations originate from floodplain and humid forests where water retention is present even during the summer months, thus providing the European wild pear with enough water. Intermediate phenotypes in the southern populations were presumably determined by climatic extremes present in this area, such as droughts, diurnal and annual temperature fluctuations and precipitation seasonality. Finally, thermophilicity of abandoned agricultural areas and high precipitation levels observed in Dinaric populations could explain their intermediate phenotypic variability.

We assume that the significant inter-population variability, noted between all of the analyzed populations, exists as a result of adaptive processes taking place under different environmental pressures [57], suggesting a close connection between plasticity and the phenotypic differences between populations and individuals [58]. Phenotypic plasticity allows an individual genotype to express different and functionally appropriate phenotypes under the influence of different habitat and environmental conditions [3], under the assumption of extensive gene flow [2]. Populations that have developed adaptive plasticity should be more responsive to environmental changes by modifying the mechanisms and traits, rather than investing in new adaptations, hence maintaining the optimal fitness of the species [58]. Such environmentally induced and non-heritable variation has previously been documented in several studies [73,74,75]. However, under the conditions of limited gene flow, such as in southern, isolated and small populations covered by this research, local adaptation is expected, rather than plasticity [2], allowing genotypes to cope with environmental heterogeneity. Nevertheless, those two mechanisms are not mutually exclusive [76], and it is expected that plant populations experience both simultaneously. European wild pear is among the species with wide ecological amplitudes, with low demand on soil conditions, tolerance to high temperatures, drought and flooding [27]. Under such diverse conditions, great phenotypic variability in this species is to be expected, taking into account plasticity and adaptability to local conditions. Accordingly, it is reasonable to assume that most of the described lower taxa are in fact only a part of intraspecific variability conditioned by local habitat and climatic conditions, additionally supported by the fact that leaf shape shows much lower variability compared to leaf size.

The results of this research showed low but statistically significant presence of both isolation by distance (IBD) and isolation by environment (IBE) patterns, with the latter being slightly more pronounced. These two models demonstrate phenotypic differentiation between populations by the homogenizing action of gene flow parallel to modifying processes such as local adaptation, plasticity, and genetic drift [77]. The isolation by distance model indicates that differentiation between populations increases with geographical distance [78], in view of limited gene flow and the presence of genetic drift [79]. On the other hand, the isolation by environment model uses environmental differences between populations to explain their phenotypic variance. In this model, low but positive correlation is present between phenotypic and environmental distances, unrelated to geographical distance [78,80]. A study by Sexton et al. [81], which analyzed the prevalence of patterns within 70 previous studies, showed that the gene flow among populations can follow both IBD and IBE patterns. In this study, we also confirmed both models influencing European wild pear population structure. However, the isolation by environment pattern proved to be more conspicuous, as correlation between phenotypic and environmental distances remained significant even after excluding the effect of geographical distance. This suggests that population phenotypic diversity in our study is influenced by climatic conditions such as temperature and its diurnal and annual range, precipitation and its seasonality and climatic extremes. Other factors, such as habitat type, microclimatic conditions, vegetation density and underlying genetic diversity of the populations are likely to play a role in the phenotypic diversity of European wild pear populations and require further research. Similar results were also confirmed for other woody species: *Alnus incana* [46], *Fraxinus angustifolia* Vahl [82], *Pyrus spinosa* [65], *Rubus idaeus* L. [83] and *Quercus acutissima* Carruth. [84], showing a unifying trend, most likely arising from a shared ability to adjust to local environments, in order to survive.

## 5. Conclusions

In this study, European wild pear populations demonstrated high variability in leaf traits at the inter- and intra-population levels. The most variable traits were those determining leaf size (leaf area, leaf width top and leaf petiole length), in contrast to the less variable leaf shape traits. Additionally, both environment and geographic distribution of populations played statistically significant roles in shaping the phenotypic variability, indicating the presence of patterns of isolation by environment (IBE) and isolation by distance (IBD). However, IBE played the greater role in shaping the phenotypic diversity in the studied populations. Accordingly, two clusters of populations emerged in the multivariate statistical analysis, with environmental factors and heterogeneous origin as the differentiators. In conclusion, we assume that the high levels of leaf trait variability observed in this study can be attributed to the pronounced phenotypic plasticity of European wild pear, as well as strong local adaptation of its populations.

## Figures and Tables

**Table 1:** Descriptive statistics for analyzed traits of *Pyrus pyraster* leaves from 19 studied populations. M—arithmetic mean; SD—standard deviation; Min—minimal value; Max—maximal value; CV—coefficient of variation (%).

Trait	Acronyms	M	SD	Min	Max	CV (%)
Leaf area (cm^2^)	LA	9.88	3.56	1.56	27.97	36.06
Perimeter (cm)	PE	11.64	2.33	5.14	21.10	20.05
Form coefficient	FC	0.90	0.12	0.51	1.44	13.70
Leaf length (cm)	LL	3.89	0.79	1.56	6.88	20.17
Maximal leaf width (cm)	MLW	3.27	0.63	1.10	5.68	19.18
Position of maximal leaf width (cm)	PMLW	1.56	0.37	0.13	2.95	23.84
Leaf width top (cm)	LWT	1.40	0.47	0.22	3.08	33.92
Leaf angle 10 (°)	LA10	69.45	5.58	34.00	79.00	8.04
Leaf angle 25 (°)	LA25	56.36	4.74	37.00	68.00	8.40
Petiole length (cm)	PL	3.54	1.15	0.61	7.95	32.50

**Table 2:** Results of the hierarchical analysis of variance for studied leaf phenotypic traits.

**Trait**	**Components of the Variance**	**df**	**F**	**Percent of Variability**	***p*** **-Value**
Leaf area (LA)	Among populations	18	5.11	14.91	<0.01
	Within populations	171	21.56	34.61	<0.01
	Error			50.48	
Perimeter (PE)	Among populations	18	4.44	12.58	<0.01
	Within populations	171	20.88	34.84	<0.01
	Error			52.58	
Form coefficient (FC)	Among populations	18	1.37	1.64	0.15
	Within populations	171	23.82	42.49	<0.01
	Error			55.87	
Leaf length (LL)	Among populations	18	3.94	11.23	<0.01
	Within populations	171	21.86	36.41	<0.01
	Error			52.36	
Maximal leaf width (MLW)	Among populations	18	5.83	17.88	<0.01
	Within populations	171	23.83	35.49	<0.01
	Error			46.63	
Position of maximal leaf width (PMLW)	Among populations	18	1.52	2.16	0.09
	Within populations	171	21.59	39.82	<0.01
	Error			58.02	
Leaf width top (LWT)	Among populations	18	3.03	9.68	<0.01
	Within populations	171	32.51	46.27	<0.01
	Error			44.05	
Leaf angle 10 (LA10)	Among populations	18	2.70	8.92	<0.01
	Within populations	171	39.66	51.28	<0.01
	Error			39.80	
Leaf angle 25 (LA25)	Among populations	18	2.88	9.38	<0.01
	Within populations	171	35.35	48.37	<0.01
	Error			42.25	
Petiole length (PL)	Among populations	18	3.62	6.86	<0.01
	Within populations	171	11.35	23.90	<0.01
	Error			69.24	

**Table 3:** Pearson’s correlation coefficients between 10 phenotypic traits and scores of the first three principal components.

Trait	PC—Principal Component		
	PC1	PC2	PC3
Leaf area (LA)	−0.979	−0.065	−0.008
Perimeter (PE)	−0.955	0.203	−0.088
Form coefficient (FC)	0.101	−0.841	0.270
Leaf length (LL)	−0.855	0.427	0.134
Maximal leaf width (MLW)	−0.923	−0.319	−0.119
Position of maximal leaf width (PMLW)	−0.758	0.534	−0.112
Leaf width top (LWT)	−0.581	−0.517	−0.360
Leaf angle 10 (LA10)	−0.166	−0.917	−0.002
Leaf angle 25 (LA25)	−0.120	−0.955	−0.161
Petiole length (PL)	−0.629	−0.195	0.682
Eigenvalue	4.81	3.38	0.75
% Total variance	48.14	33.79	7.45

## Data Availability

Not applicable.

## Supplementary Materials

The following supporting information can be downloaded at: https://www.mdpi.com/article/10.3390/plants11030335/s1, Table S1. Number of individuals (N), geographic coordinates, altitudes, and bioclimatic variables for 19 *Pyrus pyraster* populations (n = 190). Bioclim variables: BIO1 (Annual Mean Temperature); BIO2 (Mean Diurnal Range (Mean of monthly (max temp–min temp)); BIO3 (Isothermality (BIO2/BIO7) (×100)); BIO4 (Temperature Seasonality (standard deviation ×100)); BIO5 (Max Temperature of Warmest Month); BIO6 (Min Temperature of Coldest Month); BIO7 (Temperature Annual Range (BIO5-BIO6)); BIO8 (Mean Temperature of Wettest Quarter); BIO9 (Mean Temperature of Driest Quarter); BIO10 (Mean Temperature of Warmest Quarter); BIO11 (Mean Temperature of Coldest Quarter); BIO12 (Annual Precipitation); BIO13 (Precipitation of Wettest Month); BIO14 (Precipitation of Driest Month); BIO15 (Precipitation Seasonality (Coefficient of Variation)); BIO16 (Precipitation of Wettest Quarter); BIO17 (Precipitation of Driest Quarter); BIO18 (Precipitation of Warmest Quarter); BIO19 (Precipitation of Coldest Quarter). Populations: P01—Kuberton; P02—Hum; P03—Lupoglav; P04—Kozji vrh; P05—Lukovdol; P06—Ogulin; P07—Brinje; P08—Plitvička jezera; P09—Perušić; P10—Rumin; P11—Voštane; P12—Studenci; P13—Žumberak; P14—Strahinščica; P15—Kalnik; P16—Moslavačka gora; P17—Lipovljani; P18—Psunj; P19—Vinkovci; Table S2. Pearson correlation coefficients between 19 bioclimatic variables and scores of the first five principal components. Bioclim variables: BIO1 (Annual Mean Temperature); BIO2 (Mean Diurnal Range (Mean of monthly (max temp–min temp)); BIO3 (Isothermality (BIO2/BIO7) (×100)); BIO4 (Temperature Seasonality (standard deviation ×100)); BIO5 (Max Temperature of Warmest Month); BIO6 (Min Temperature of Coldest Month); BIO7 (Temperature Annual Range (BIO5–BIO6)); BIO8 (Mean Temperature of Wettest Quarter); BIO9 (Mean Temperature of Driest Quarter); BIO10 (Mean Temperature of Warmest Quarter); BIO11 (Mean Temperature of Coldest Quarter); BIO12 (Annual Precipitation); BIO13 (Precipitation of Wettest Month); BIO14 (Precipitation of Driest Month); BIO15 (Precipitation Seasonality (Coefficient of Variation)); BIO16 (Precipitation of Wettest Quarter); BIO17 (Precipitation of Driest Quarter); BIO18 (Precipitation of Warmest Quarter); BIO19 (Precipitation of Coldest Quarter); Figure S1. Biplot of the principal component (PC) analysis based on 19 bioclimatic variables in 19 studied *Pyrus pyraster* populations. Bioclim variables: BIO1 (Annual Mean Temperature); BIO2 (Mean Diurnal Range (Mean of monthly (max temp–min temp)); BIO3 (Isothermality (BIO2/BIO7) (×100)); BIO4 (Temperature Seasonality (standard deviation ×100)); BIO5 (Max Temperature of Warmest Month); BIO6 (Min Temperature of Coldest Month); BIO7 (Temperature Annual Range (BIO5-BIO6)); BIO8 (Mean Temperature of Wettest Quarter); BIO9 (Mean Temperature of Driest Quarter); BIO10 (Mean Temperature of Warmest Quarter); BIO11 (Mean Temperature of Coldest Quarter); BIO12 (Annual Precipitation); BIO13 (Precipitation of Wettest Month); BIO14 (Precipitation of Driest Month); BIO15 (Precipitation Seasonality (Coefficient of Variation)); BIO16 (Precipitation of Wettest Quarter); BIO17 (Precipitation of Driest Quarter); BIO18 (Precipitation of Warmest Quarter); BIO19 (Precipitation of Coldest Quarter). Populations: P01—Kuberton; P02—Hum; P03—Lupoglav; P04—Kozji vrh; P05—Lukovdol; P06—Ogulin; P07—Brinje; P08—Plitvička jezera; P09—Perušić; P10—Rumin; P11—Voštane; P12—Studenci; P13—Žumberak; P14—Strahinščica; P15—Kalnik; P16—Moslavačka gora; P17—Lipovljani; P18—Psunj; P19—Vinkovci; Table S3. Descriptive statistics shown for each studied population. Populations: P01—Kuberton; P02 —Hum; P03—Lupoglav; P04—Kozji vrh; P05—Lukovdol; P06—Ogulin; P07—Brinje; P08—Plitvička jezera; P09—Perušić; P10—Rumin; P11—Voštane; P12—Studenci; P13—Žumberak; P14—Strahinščica; P15—Kalnik; P16—Moslavačka gora; P17—Lipovljani; P18—Psunj; P19—Vinkovci. Leaf phenotypic traits: LA—leaf area; PE—perimeter; LL—leaf length; MLW—maximum leaf width; PMLW—leaf length, measured from the leaf base to the point of maximum leaf width; LWT—leaf blade width at 90% of leaf blade length; PL—petiole length; FC—form coefficient; LA10—angle closed by the main leaf vein (the center of the leaf blade) and the line connecting the leaf blade base to a set point on the leaf margin at 10% of total leaf blade length; and LA25—angle closed by the main leaf vein (the center of the leaf blade) and the line connecting the leaf blade base to a set point on the leaf margin at 25% of total leaf blade length. Maximal and minimal values for arithmetic mean (M) and coefficient of variation (CV) are highlighted with red and green colors, respectively; Table S4. Results of the stepwise discriminant analyses for studied morphometric traits; Table S5. Correlations between geographic, bioclimatic and phenotypic variables. Statistically significant values are highlighted with red color. Bioclim variables: BIO1 (Annual Mean Temperature); BIO2 (Mean Diurnal Range (Mean of monthly (max temp–min temp)); BIO3 (Isothermality (BIO2/BIO7) (×100)); BIO4 (Temperature Seasonality (standard deviation ×100)); BIO5 (Max Temperature of Warmest Month); BIO6 (Min Temperature of Coldest Month); BIO7 (Temperature Annual Range (BIO5-BIO6)); BIO8 (Mean Temperature of Wettest Quarter); BIO9 (Mean Temperature of Driest Quarter); BIO10 (Mean Temperature of Warmest Quarter); BIO11 (Mean Temperature of Coldest Quarter); BIO12 (Annual Precipitation); BIO13 (Precipitation of Wettest Month); BIO14 (Precipitation of Driest Month); BIO15 (Precipitation Seasonality (Coefficient of Variation)); BIO16 (Precipitation of Wettest Quarter); BIO17 (Precipitation of Driest Quarter); BIO18 (Precipitation of Warmest Quarter); BIO19 (Precipitation of Coldest Quarter). Leaf phenotypic traits: LA–leaf area; PE—perimeter; LL—leaf length; MLW—maximum leaf width; PMLW—leaf length, measured from the leaf base to the point of maximum leaf width; LWT—leaf blade width at 90% of leaf blade length; PL—petiole length; FC—form coefficient; LA10—angle closed by the main leaf vein (the center of leaf blade) and the line connecting the leaf blade base to a set point on the leaf margin at 10% of total leaf blade length; and LA25—angle closed by the main leaf vein (the center of leaf blade) and the line connecting the leaf blade base to a set point on the leaf margin at 25% of total leaf blade length.
