# Supplementary material for: Phenotypic Variation in European Wild Pear (Pyrus pyraster (L.) Burgsd.) Populations in the North-Western Part of the Balkan Peninsula
Source: Plants (Basel). 2022 Jan 27;11(3):335. doi: 10.3390/plants11030335 (PMC8837925; doi:10.3390/plants11030335)
Supplement: Supplementary file 1 [file plants-11-00335-s001.zip › plants-1564938-supplementary.pdf]

Table S1. Number of individuals (N), geographic coordinates, altitudes, and bioclimatic variables for 19 *Pyrus pyraster* populations (n = 190). Bioclim variables: BIO1 (Annual Mean Temperature); BIO2 (Mean Diurnal Range (Mean of monthly (max temp–min temp)); BIO3 (Isothermality (BIO2/BIO7) (×100)); BIO4 (Temperature Seasonality (standard deviation ×100)); BIO5 (Max Temperature of Warmest Month); BIO6 (Min Temperature of Coldest Month); BIO7 (Temperature Annual Range (BIO5-BIO6)); BIO8 (Mean Temperature of Wettest Quarter); BIO9 (Mean Temperature of Driest Quarter); BIO10 (Mean Temperature of Warmest Quarter); BIO11 (Mean Temperature of Coldest Quarter); BIO12 (Annual Precipitation); BIO13 (Precipitation of Wettest Month); BIO14 (Precipitation of Driest Month); BIO15 (Precipitation Seasonality (Coefficient of Variation)); BIO16 (Precipitation of Wettest Quarter); BIO17 (Precipitation of Driest Quarter); BIO18 (Precipitation of Warmest Quarter); BIO19 (Precipitation of Coldest Quarter). Populations: P01—Kuberton; P02—Hum; P03—Lupoglav; P04—Kozji vrh; P05—Lukovdol; P06—Ogulin; P07—Brinje; P08—Plitvička jezera; P09—Perušić; P10—Rumin; P11—Voštane; P12—Studenci; P13—Žumberak; P14—Strahinščica; P15—Kalnik; P16—Moslavačka gora; P17—Lipovljani; P18—Psunj; P19—Vinkovci.

Pop. ID	N	Longitude	Latitude	Alt.	Bioclim variables																		
					1	2	3	4	5	6	7	8	9	10	11	12	13	14	15	16	17	18	19
P01	10	13.777	45.433	440	11.8	8.3	32.5	649.1	24.3	-1.2	25.5	8.2	4.9	20.1	4.2	1272	143	72	19.9	400	259	293	285
P02	10	14.046	45.351	304	12.4	9.4	34.3	664.9	25.7	-1.6	27.3	8.6	5.4	20.9	4.5	1190	138	70	21.1	385	250	268	276
P03	10	14.094	45.368	391	10.6	8.7	34.2	635.1	22.9	-2.6	25.5	7.2	3.7	18.7	3.3	1380	164	82	20.9	448	293	308	325
P04	10	14.591	45.628	885	7.0	9.2	33.8	684.3	20.0	-7.2	27.2	7.9	-0.4	15.6	-1.1	1505	163	89	18.1	452	293	387	302
P05	10	15.123	45.434	400	9.6	9.2	32.1	690.4	23.0	-5.5	28.5	9.9	2.4	18.1	1.1	1428	155	78	19.9	440	263	364	283
P06	10	15.244	45.264	320	10.3	9.8	33.0	717.4	24.0	-5.6	29.6	10.5	2.8	19.0	1.3	1331	146	79	19.2	418	257	321	276
P07	10	15.199	45.059	496	8.7	11.2	36.9	711.8	22.8	-7.5	30.3	9.1	1.4	17.5	-0.2	1382	165	81	22.6	455	287	290	314
P08	10	15.687	44.858	646	8.6	10.1	34.7	678.4	22.3	-6.9	29.2	9.3	1.3	17.0	0.3	1340	157	86	19.5	426	284	289	308
P09	10	15.345	44.672	572	8.9	10.8	35.4	718.3	23.2	-7.2	30.4	4.5	17.8	17.8	0.03	1246	162	58	28.1	432	225	225	311
P10	10	16.708	43.826	938	8.4	12.0	40.4	662.4	22.8	-7.0	29.8	5.1	16.6	16.6	0.4	1045	134	54	25.9	361	199	199	265
P11	10	16.900	43.668	1026	7.4	11.8	39.9	651.1	21.9	-7.7	29.6	4.5	15.6	15.6	-0.3	1039	135	52	26.5	361	194	194	267
P12	10	17.039	43.537	668	10.5	10.9	38.2	654.2	24.0	-4.5	28.5	7.3	18.7	18.7	2.6	963	128	45	29.1	344	169	169	257
P13	10	15.533	45.818	663	8.7	8.6	30.8	702.6	21.9	-6.2	28.1	17.4	1.4	17.4	0.1	1207	132	59	21.8	358	209	358	215
P14	10	15.916	46.195	543	9.0	8.6	30.2	715.7	22.4	-6.2	28.6	17.8	1.6	17.8	0.2	1107	127	52	24.2	348	187	348	190
P15	10	16.530	46.157	280	10.1	8.7	29.7	723.6	23.7	-5.5	29.2	18.9	2.6	18.9	0.9	852	96	43	23.3	264	142	264	150
P16	10	16.704	45.566	107	11.3	9.9	31.4	768.9	26.0	-5.5	31.5	20.7	3.4	20.7	1.7	889	92	51	17.7	256	165	256	173
P17	10	16.829	45.375	105	11.2	9.9	32.3	731.0	25.5	-5.4	30.9	20.0	3.6	20.0	1.9	918	93	54	16.1	263	175	263	183
P18	10	17.312	45.338	531	9.6	8.3	30.7	688.4	22.0	-5.1	27.1	17.9	2.3	17.9	0.9	995	111	57	18.6	300	185	300	193
P19	10	18.874	45.236	96	11.1	9.9	31.5	785.3	25.4	-5.9	31.3	20.6	2.9	20.6	1.2	678	87	39	22.9	218	125	218	136

Table S2. Pearson correlation coefficients between 19 bioclimatic variables and scores of the first five principal components. Bioclim variables: BIO1 (Annual Mean Temperature); BIO2 (Mean Diurnal Range (Mean of monthly (max temp–min temp)); BIO3 (Isothermality (BIO2/BIO7) (×100)); BIO4 (Temperature Seasonality (standard deviation ×100)); BIO5 (Max Temperature of Warmest Month); BIO6 (Min Temperature of Coldest Month); BIO7 (Temperature Annual Range (BIO5–BIO6)); BIO8 (Mean Temperature of Wettest Quarter); BIO9 (Mean Temperature of Driest Quarter); BIO10 (Mean Temperature of Warmest Quarter); BIO11 (Mean Temperature of Coldest Quarter); BIO12 (Annual Precipitation); BIO13 (Precipitation of Wettest Month); BIO14 (Precipitation of Driest Month); BIO15 (Precipitation Seasonality (Coefficient of Variation)); BIO16 (Precipitation of Wettest Quarter); BIO17 (Precipitation of Driest Quarter); BIO18 (Precipitation of Warmest Quarter); BIO19 (Precipitation of Coldest Quarter).

Variable	PC—Principal Component				
	PC1	PC2	PC3	PC4	PC5
BIO1	0.586	-0.503	-0.601	-0.189	-0.057
BIO2	-0.165	0.861	-0.188	-0.396	0.170
BIO3	-0.487	0.698	-0.422	-0.125	0.248
BIO4	0.681	0.026	0.494	-0.447	-0.262
BIO5	0.704	-0.154	-0.523	-0.442	-0.050
BIO6	0.146	-0.667	-0.702	0.191	0.013
BIO7	0.466	0.576	0.287	-0.596	-0.058
BIO8	0.816	-0.301	0.462	0.032	0.027
BIO9	-0.088	0.767	-0.548	0.135	-0.111
BIO10	0.684	-0.469	-0.461	-0.277	-0.140
BIO11	0.247	-0.508	-0.821	0.020	0.046
BIO12	-0.904	-0.355	0.074	-0.164	-0.116
BIO13	-0.963	-0.073	-0.091	-0.114	-0.206
BIO14	-0.795	-0.488	0.072	-0.316	0.123
BIO15	-0.102	0.744	-0.314	0.298	-0.465
BIO16	-0.955	-0.146	-0.088	-0.146	-0.184
BIO17	-0.874	-0.398	-0.051	-0.262	0.032
BIO18	-0.348	-0.713	0.557	0.053	-0.108
BIO19	-0.924	-0.019	-0.302	-0.208	-0.039
Eigenvalue	8.05	5.05	3.60	1.46	0.54
% Total Variance	42.36	26.58	18.94	7.66	2.83

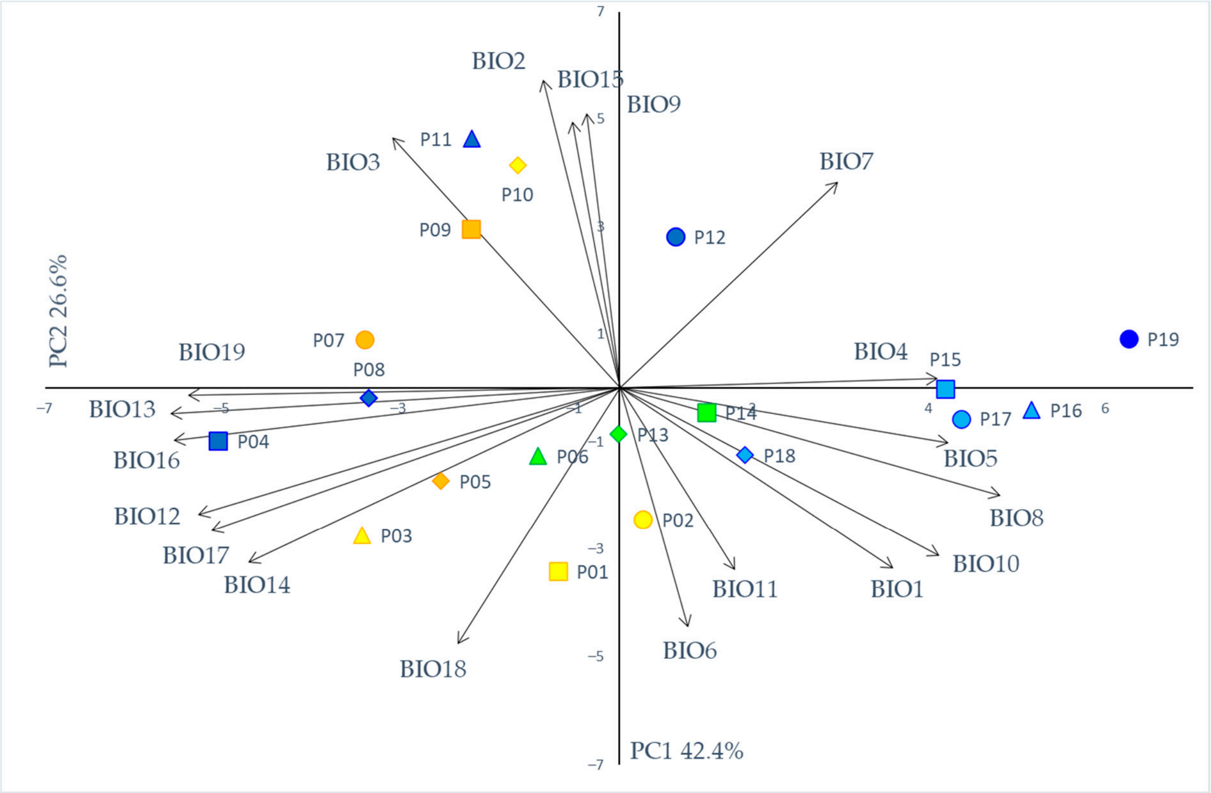

Figure S1. Biplot of the principal component (PC) analysis based on 19 bioclimatic variables in 19 studied *Pyrus pyraster* populations. Bioclim variables: BIO1 (Annual Mean Temperature); BIO2 (Mean Diurnal Range (Mean of monthly (max temp–min temp)); BIO3 (Isothermality (BIO2/BIO7) (×100)); BIO4 (Temperature Seasonality (standard deviation ×100)); BIO5 (Max Temperature of Warmest Month); BIO6 (Min Temperature of Coldest Month); BIO7 (Temperature Annual Range (BIO5-BIO6)); BIO8 (Mean Temperature of Wettest Quarter); BIO9 (Mean Temperature of Driest Quarter); BIO10 (Mean Temperature of Warmest Quarter); BIO11 (Mean Temperature of Coldest Quarter); BIO12 (Annual Precipitation); BIO13 (Precipitation of Wettest Month); BIO14 (Precipitation of Driest Month); BIO15 (Precipitation Seasonality (Coefficient of Variation)); BIO16 (Precipitation of Wettest Quarter); BIO17 (Precipitation of Driest Quarter); BIO18 (Precipitation of Warmest Quarter); BIO19 (Precipitation of Coldest Quarter). Populations: P01—Kuberton; P02—Hum; P03—Lupoglav; P04—Kozji vrh; P05—Lukovdol; P06—Ogulin; P07—Brinje; P08—Plitvička jezera; P09—Perušić; P10—Rumin; P11—Voštane; P12—Studenci; P13—Žumberak; P14—Strahinščica; P15—Kalnik; P16—Moslavačka gora; P17—Lipovljani; P18—Psunj; P19—Vinkovci.

Table S3. Descriptive statistics shown for each studied population. Populations: P01—Kuberton; P02—Hum; P03—Lupoglav; P04—Kozji vrh; P05—Lukovdol; P06—Ogulin; P07—Brinje; P08—Plitvička jezera; P09—Perušić; P10—Rumin; P11—Voštane; P12—Studenci; P13—Žumberak; P14—Strahinščica; P15—Kalnik; P16—Moslavačka gora; P17—Lipovljani; P18—Psunj; P19—Vinkovci. Leaf phenotypic traits: LA—leaf area; PE—perimeter; LL—leaf length; MLW—maximum leaf width; PMLW—leaf length, measured from the leaf base to the point of maximum leaf width; LWT—leaf blade width at 90% of leaf blade length; PL—petiole length; FC—form coefficient; LA10—angle closed by the main leaf vein (the center of the leaf blade) and the line connecting the leaf blade base to a set point on the leaf margin at 10% of total leaf blade length; and LA25—angle closed by the main leaf vein (the center of the leaf blade) and the line connecting the leaf blade base to a set point on the leaf margin at 25% of total leaf blade length. Maximal and minimal values for arithmetic mean (M) and coefficient of variation (CV) are highlighted with red and green colors, respectively.

Population	LA		PE		FC		LL		MLW		PMLW		LWT		LA10		LA25		PL	
	M	CV	M	CV	M	CV	M	CV	M	CV	M	CV	M	CV	M	CV	M	CV	M	CV
P01	7.40	27.10	10.45	16.81	0.85	13.13	3.64	18.16	2.73	16.17	1.54	22.13	1.14	36.89	65.13	10.34	52.31	11.22	3.36	31.80
P02	7.29	35.78	9.87	18.60	0.92	12.70	3.43	19.48	2.79	20.02	1.38	26.85	1.15	37.56	68.19	9.39	55.27	9.98	2.86	33.44
P03	7.01	43.31	9.91	20.60	0.86	12.71	3.50	19.66	2.61	23.25	1.42	23.53	1.04	42.51	63.24	14.35	52.18	9.73	3.09	37.38
P04	10.00	34.74	11.66	19.81	0.91	12.72	3.85	19.85	3.34	17.32	1.64	23.19	1.52	33.17	69.36	7.71	56.84	7.93	3.84	28.44
P05	9.57	31.66	11.23	19.63	0.95	14.13	3.93	18.75	3.15	16.70	1.47	26.33	1.24	28.53	70.13	6.25	55.88	6.97	3.49	28.65
P06	10.12	41.68	11.64	22.35	0.91	12.06	3.71	20.04	3.42	22.46	1.50	26.98	1.57	36.48	71.74	5.02	58.79	5.85	3.43	39.40
P07	8.73	30.27	11.13	20.74	0.89	15.51	3.57	19.58	3.18	14.50	1.45	25.42	1.37	29.50	70.99	6.38	58.11	7.00	3.47	27.95
P08	9.93	26.51	11.73	15.56	0.90	11.54	3.88	15.04	3.32	14.00	1.57	20.65	1.46	26.00	69.84	5.82	56.88	5.59	3.81	24.69
P09	9.92	36.55	11.37	19.29	0.96	11.69	3.88	18.81	3.28	19.35	1.56	23.12	1.45	32.62	69.63	6.83	56.56	7.32	3.60	29.78
P10	8.59	28.28	11.09	15.74	0.87	11.38	3.57	17.67	3.11	13.66	1.58	19.87	1.55	25.02	68.81	6.79	56.49	7.21	3.22	28.74
P11	10.15	28.89	11.76	18.23	0.92	15.38	3.79	17.55	3.39	14.93	1.55	22.08	1.64	24.71	71.11	5.90	58.03	6.16	3.79	24.82
P12	11.25	24.85	12.36	13.54	0.92	11.77	4.21	13.22	3.42	15.14	1.76	17.75	1.63	23.31	69.19	5.79	55.35	7.07	3.60	27.57
P13	9.99	32.44	11.73	18.38	0.91	15.43	3.97	19.24	3.28	17.60	1.54	29.26	1.32	39.03	69.69	9.95	56.18	10.00	3.73	31.69
P14	10.00	36.10	11.65	20.67	0.91	13.74	3.75	21.30	3.42	17.47	1.50	22.15	1.45	28.28	71.46	5.25	58.69	6.17	3.08	38.81
P15	12.32	35.73	13.29	19.68	0.86	13.07	4.30	19.88	3.71	18.38	1.61	23.98	1.45	31.40	71.68	4.16	57.86	5.64	3.27	34.08
P16	11.99	34.82	13.10	17.21	0.86	11.62	4.34	18.10	3.64	17.68	1.67	19.30	1.36	33.15	69.96	5.55	56.71	6.02	3.54	36.21
P17	11.98	30.04	12.95	18.15	0.90	15.33	4.54	18.71	3.48	15.42	1.63	23.98	1.18	34.88	69.86	6.58	55.07	8.60	4.32	28.09
P18	10.43	29.38	11.84	17.45	0.93	13.85	3.94	19.35	3.41	15.84	1.65	23.77	1.63	28.86	69.44	9.14	56.81	9.36	3.88	28.62
P19	11.08	25.36	12.39	16.20	0.91	14.21	4.17	19.32	3.51	12.74	1.64	19.26	1.36	31.83	70.01	6.63	56.77	8.77	3.94	30.77

Table S4. Results of the stepwise discriminant analyses for studied morphometric traits.

Trait	Wilks'	Partial	F-remove	*p*-value
LA–leaf area	0.123	0.744	3.115	0.0001
FC–Form coefficient	0.104	0.886	1.171	0.2910
LL–leaf length	0.110	0.835	1.794	0.0298
MLW–maximum leaf width	0.109	0.843	1.683	0.0471
PMLW–leaf length, measured from the leaf base to the point of maximum leaf width	0.100	0.921	0.772	0.7303
LWT–leaf width top	0.109	0.839	1.738	0.0385
LA10–leaf angle 10	0.110	0.837	1.766	0.0341
LA25–leaf angle 25	0.109	0.841	1.710	0.0428
PL–petiole length	0.123	0.746	3.088	0.0001

Table S5. Correlations between geographic, bioclimatic and phenotypic variables. Statistically significant values are highlighted with red color. Bioclim variables: BIO1 (Annual Mean Temperature); BIO2 (Mean Diurnal Range (Mean of monthly (max temp–min temp)); BIO3 (Isothermality (BIO2/BIO7) (×100)); BIO4 (Temperature Seasonality (standard deviation ×100)); BIO5 (Max Temperature of Warmest Month); BIO6 (Min Temperature of Coldest Month); BIO7 (Temperature Annual Range (BIO5-BIO6)); BIO8 (Mean Temperature of Wettest Quarter); BIO9 (Mean Temperature of Driest Quarter); BIO10 (Mean Temperature of Warmest Quarter); BIO11 (Mean Temperature of Coldest Quarter); BIO12 (Annual Precipitation); BIO13 (Precipitation of Wettest Month); BIO14 (Precipitation of Driest Month); BIO15 (Precipitation Seasonality (Coefficient of Variation)); BIO16 (Precipitation of Wettest Quarter); BIO17 (Precipitation of Driest Quarter); BIO18 (Precipitation of Warmest Quarter); BIO19 (Precipitation of Coldest Quarter). Leaf phenotypic traits: LA–leaf area; PE—perimeter; LL—leaf length; MLW—maximum leaf width; PMLW—leaf length, measured from the leaf base to the point of maximum leaf width; LWT—leaf blade width at 90% of leaf blade length; PL—petiole length; FC—form coefficient; LA10—angle closed by the main leaf vein (the center of leaf blade) and the line connecting the leaf blade base to a set point on the leaf margin at 10% of total leaf blade length; and LA25—angle closed by the main leaf vein (the center of leaf blade) and the line connecting the leaf blade base to a set point on the leaf margin at 25% of total leaf blade length.

Variable	LA	PE	FC	LL	MLW	PMLW	LWT	LA10	LA25	PL
Latitude	0.399	0.316	0.381	0.210	0.458	0.030	0.291	0.501	0.548	0.110
Longitude	-0.274	-0.185	-0.399	-0.088	-0.337	0.070	-0.261	-0.431	-0.497	-0.032
BIO1	-0.062	-0.046	-0.309	0.180	-0.216	-0.065	-0.583	-0.361	-0.503	-0.221
BIO2	0.077	0.086	0.135	-0.053	0.153	0.144	0.430	0.285	0.306	0.149
BIO3	-0.304	-0.294	0.073	-0.379	-0.247	-0.021	0.330	-0.085	-0.003	-0.062
BIO4	0.656	0.655	0.052	0.592	0.684	0.293	0.037	0.555	0.469	0.344
BIO5	0.160	0.180	-0.254	0.330	0.040	0.039	-0.419	-0.073	-0.241	-0.095
BIO6	-0.474	-0.459	-0.340	-0.211	-0.621	-0.274	-0.634	-0.705	-0.755	-0.446
BIO7	0.647	0.649	0.139	0.517	0.699	0.327	0.307	0.688	0.593	0.392
BIO8	0.622	0.646	-0.171	0.658	0.574	0.327	-0.114	0.328	0.209	0.304
BIO9	-0.019	-0.048	0.202	-0.048	-0.039	0.246	0.376	-0.051	-0.067	-0.060
BIO10	0.064	0.078	-0.275	0.275	-0.071	-0.018	-0.551	-0.228	-0.373	-0.157
BIO11	-0.394	-0.377	-0.339	-0.127	-0.555	-0.224	-0.612	-0.637	-0.723	-0.404
BIO12	-0.618	-0.647	0.158	-0.627	-0.563	-0.574	-0.200	-0.265	-0.152	-0.247
BIO13	-0.684	-0.718	0.226	-0.727	-0.606	-0.552	-0.069	-0.291	-0.139	-0.308
BIO14	-0.587	-0.594	0.012	-0.567	-0.545	-0.538	-0.275	-0.309	-0.193	-0.151
BIO15	-0.033	-0.062	0.249	-0.162	0.030	0.088	0.408	0.121	0.171	-0.263
BIO16	-0.680	-0.713	0.204	-0.717	-0.609	-0.578	-0.105	-0.280	-0.143	-0.326
BIO17	-0.704	-0.713	0.029	-0.677	-0.660	-0.611	-0.295	-0.386	-0.256	-0.253
BIO18	-0.202	-0.221	0.067	-0.204	-0.166	-0.387	-0.289	-0.027	0.021	-0.080
BIO19	-0.699	-0.723	0.140	-0.691	-0.662	-0.487	-0.102	-0.396	-0.272	-0.264
